# Diarrhoea and preadmission antibiotic exposure in COVID-19: a retrospective cohort study of 1153 hospitalised patients

**DOI:** 10.1136/bmjgast-2020-000593

**Published:** 2021-09-06

**Authors:** Bilal Akhter Mateen, Sandip Samanta, Sebastian Tullie, Sarah O’Neill, Zillah Cargill, Gillian Kelly, Ewen Brennan, Mehul Patel, Mohammad Al-Agil, James Galloway, James Teo, Debbie L Shawcross, Alexandra J Kent, Bu'Hussain Hayee

**Affiliations:** 1 Institute of Health Informatics, University College London, London, UK; 2 King's College Hospital NHS Foundation Trust, London, UK; 3 School of Immunology & Microbial Sciences, King's College London, London, UK

**Keywords:** diarrhoea, COVID-19, antibiotic therapy

## Abstract

**Objective:**

The aims of this study were to describe community antibiotic prescribing patterns in individuals hospitalised with COVID-19, and to determine the association between experiencing diarrhoea, stratified by preadmission exposure to antibiotics, and mortality risk in this cohort.

**Design/methods:**

Retrospective study of the index presentations of 1153 adult patients with COVID-19, admitted between 1 March 2020 and 29 June 2020 in a South London NHS Trust. Data on patients’ medical history (presence of diarrhoea, antibiotic use in the previous 14 days, comorbidities); demographics (age, ethnicity, and body mass index); and blood test results were extracted. Time to event modelling was used to determine the risk of mortality for patients with diarrhoea and/or exposure to antibiotics.

**Results:**

19.2% of the cohort reported diarrhoea on presentation; these patients tended to be younger, and were less likely to have recent exposure to antibiotics (unadjusted OR 0.64, 95% CI 0.42 to 0.97). 19.1% of the cohort had a course of antibiotics in the 2 weeks preceding admission; this was associated with dementia (unadjusted OR 2.92, 95% CI 1.14 to 7.49). After adjusting for confounders, neither diarrhoea nor recent antibiotic exposure was associated with increased mortality risk. However, the absence of diarrhoea in the presence of recent antibiotic exposure was associated with a 30% increased risk of mortality.

**Conclusion:**

Community antibiotic use in patients with COVID-19, prior to hospitalisation, is relatively common, and absence of diarrhoea in antibiotic-exposed patients may be associated with increased risk of mortality. However, it is unclear whether this represents a causal physiological relationship or residual confounding.

**Summary box**

**What is already known about this subject?**

- Several meta-analyses have identified that while diarrhoeal symptoms are prevalent in between 5% and 20% of patients with COVID-19, there was no discernible association with increased mortality risk. There is only one other study to date reporting on prehospital use of antibiotics and enteric symptoms. The study in question has several limitations, most notably not including postdischarge events, and failing to account for informative censoring (ie, discharge).

**What are the new findings?**

- The core result of this study is concordant with previous meta-analyses, after adjusting for confounders, neither diarrhoea nor exposure to antibiotics is independently associated with mortality in COVID-19. However, we contribute evidence of substantial prehospital use of antibiotics (ie, in up to 20% of all admissions). Moreover, absence of diarrhoea in the presence of recent antibiotic exposure appears to be associated with increased risk of mortality; however, a mechanistic explanation is beyond the scope of the analysis.

**How might it impact on clinical practice in the foreseeable future?**

- Demonstrations of a potential interaction between antibiotic exposure and diarrhoea (or the lack thereof) highlight the need for further research on the gut-lung axis to facilitate a better understanding of the impacts of antibiotics on host physiology. If a causal relationship exists, and these results are not the effect of residual confounding, the implications would suggest that antimicrobial stewardship has a second important role in contributing to patients’ health and well-being, alongside mitigating the risk of antimicrobial resistance.

## Introduction

Since the advent of COVID-19, caused by the highly infectious SARS-CoV-2,^1^ there have been reports of a clinical phenotype including gastrointestinal (GI) symptoms.^2^ Systematic reviews of the literature suggest that 5%–20% of individuals with COVID-19 experience diarrhoea,^3–5^ likely due to concomitant GI infection (viral RNA is detectable in approximately 50% of affected patients).^6^ Patients with this clinical phenotype appear to take longer to present to healthcare services, and to test positive for COVID-19.^7^ However, the aforementioned meta-analyses, which largely comprised early studies from China, suggest that overall mortality risk was not significantly increased in this subgroup.^3 8^ A limitation of these reviews is that they fail to account for the impact of a key confounder on the association between diarrhoea and mortality risk: that of antibiotic exposure.

Human coronavirus infections increase the risk of pneumococcal adherence to local epithelia and thus increase the risk of secondary bacterial pneumonia.^9^ With epidemiological evidence of the impact of secondary bacterial pneumonias during the 2003 SARS outbreak and several influenza pandemics,^10 11^ antibiotics were included as part of treatment recommendations for some symptomatic patients in the UK.^12^ Notably, diarrhoea is a common side effect of antibiotics with frequency often being agent specific, but generally occurring in 5%–35% of patients.^13^ As such, it is possible that the GI phenotype of COVID-19 reported includes an unrecognised subgroup where diarrhoea was iatrogenic and not driven by SARS-CoV-2. The biological plausibility of the latter (ie, SARS-CoV-2-driven enteric symptoms) is already well established as both major cell receptors that SARS-CoV-2 uses to enter hosts cell (ie, ACE2 and the transmembrane serine protease 2) are highly expressed by enterocytes in the ileum and colon.^14^

We conducted this study using data from two hospital sites in London, the most heavily impacted region of the UK in terms of absolute number of cases,^15^ to describe the extent to which antibiotic therapy was initiated in the community for individuals who were subsequently hospitalised and found to be COVID-19 positive; and to determine the association between experiencing diarrhoea, stratified by preadmission exposure to antibiotics, and mortality risk in this cohort.

## Materials and methods

### Data source

Data were extracted from the electronic health records system used at King’s College Hospital NHS Foundation Trust (Sunrise Clinical Manager, Allscripts), which is operated at both of its constituent sites (King’s College Hospital and Princess Royal University Hospital), servicing a total population of approximately 1.2 million people in South London.

### Study population

Information was extracted for all patients admitted via the emergency department with a positive reverse transcription PCR oronasopharyngeal swab for SARS-CoV-2 between 1 March 2020 and 29 May 2020. Additional details on the testing procedures in England are detailed in the online supplemental material.

10.1136/bmjgast-2020-000593.supp1 Supplementary data

All patients over 18 years old whose first positive oronasopharyngeal swab was taken within 72 hours of admission were included. To ensure the cohort adequately reflected the experiences of community-dwelling individuals with COVID-19 admitted on their index presentation, the following exclusion criteria were applied: (1) individuals who presented to an emergency department but were not admitted in the 14 days prior to their subsequent admission (as their subsequent admission was not their true index presentation); (2) individuals with a known positive test in the community, or from another hospital (the latter implies there was an admission as national guidance suggested testing only if the patient was due to be admitted); (3) individuals transferred from another inpatient facility (ie, inpatient psychiatry or rehabilitation); (4) individuals who were incidentally admitted during this period (eg, for traumatic injuries), and were found to be asymptomatic cases of COVID-19; (5) patients admitted to any hospital in the last 14 days due to the risk of nosocomial infection or treatment-related enteric symptoms.

### Recorded clinical features

The data specification for each patient comprised: demographics (self-identified gender and ethnicity, age, and Index of Multiple Deprivation (IMD)), body mass index (BMI), comorbidities, severity markers (blood test results), and information on the exposures of interest. Ethnicity was coded according to the UK census groups, that is, white, black, Asian, mixed, other, or missing. Each patient’s postal code was linked to the corresponding 2019 IMD Score^16^; an area-level composite score of socioeconomic status. BMI was recoded from continuous form into categories based on the WHO classification: <18.5, 18.5–24.9, 25–29.9, 30–39.9, >39.9, or missing. Comorbidities included: hypertension, ischaemic heart disease, congestive heart failure, cerebrovascular disease, dementia, chronic pulmonary disease (predominantly asthma, chronic obstructive pulmonary disease and interstitial lung disease), diabetes (type 1 and type 2), chronic kidney disease, previous or active cancer, and chronic liver disease (excluding explicitly diagnosed mild disease). The following blood test results from samples taken during the emergency department assessment were also extracted from the electronic health records: C-reactive protein, urea, creatinine, platelet count, neutrophil count, and lymphocyte count. Moreover, initial National Early Warning Score 2 result on presentation to the emergency department was extracted,^17^ as were reports of diarrhoea (documented in the emergency department review, admission clerking, or any entry from the first 24 hours of the admission, and reflecting either a patient-reported phenomenon, or objective finding of Bristol type 6 or 7 stool alongside increased frequency recorded on a stool chart by a healthcare professional).^18^ Finally, any records of antibiotics being prescribed in the 14 days preceding admission were extracted from the electronic health records. Further details on the extraction method can be found in the online supplemental material.

### Outcome

The primary outcome was all-cause mortality. Patients were followed up from hospital admission to either the earliest of death or the end of the follow-up period. Discharge was not treated as a competing risk event given that both post-transfer and postdischarge mortality data were captured. To ensure adequate follow-up, outcomes were manually extracted on 30 July 2020, such that everyone had the potential for at least 62 days from inclusion. Secondary outcomes of admission to a critical care unit and mechanical ventilation were also extracted. However, the secondary outcomes are not used in any modelling-based analysis due to insufficient event numbers. A set of sensitivity analyses based on censoring at 30 days of follow-up is described in the online supplemental sTable 4 and sFigure 2, as evidence that the extensive follow-up did not obscure/bias any potential effects observable shortly after contracting COVID-19.

### Statistical analysis

Descriptive (numerical) summaries for the recorded clinical features are presented as follows: continuous data are presented as median and range, and categorical data are presented as absolute counts (ie, frequency) with proportions, for the entire cohort and stratified by presence of diarrhoea and/or exposure to antibiotics. Statistical hypothesis testing was conducted using either the Student’s t-test, Wilcoxon rank-sum test, or χ^2^ test. The method described by Benjamini-Hochberg was used for multiple testing correction. The threshold for significance was set at 5% for all tests. All analyses were carried out using R (V.3.6.2), and the following packages: survival, rms, coxme, and mice.

#### Primary analysis

Time to event (survival) modelling was used to determine the risk of mortality for each cohort (presence of diarrhoea and/or exposure to antibiotics). Kaplan-Meier survival functions were used to visualise the univariable and subgroup effects, with Cox proportional hazards (CPH) models used to address confounding through adjustment for other relevant measurements. For each model the satisfaction of the proportional hazards assumption was assessed through examination of the Schoenfeld residuals.

Multivariable CPH models were developed through sequential adjustment for risk factors, starting with a univariate model composed just of the exposure of interest (ie, presence of diarrhoea, exposure to antibiotics, or the diarrhoea:antibiotics interaction term). The first adjustment in the prespecified sequence was for age, then gender, then IMD, followed by the comorbidities, and finally the severity markers; the primary fully adjusted model was composed of all the aforementioned variables. Due to the expected degree of missingness in the ethnicity and BMI variables, these were not planned to form part of the core model. These data are unlikely to be amenable to best practice missing data handling procedures such as multiple imputation as it is a priori difficult to justify a missingness at random assumption. Therefore, adjustments for ethnicity and BMI were done following completion of the core model, with a missingness indicator. Prior studies have demonstrated that using such an approach will likely bias the effect of interest, and thus all results are interpreted in light of this.^19 20^ For all continuous variables, a comparison of a linear continuous term, a categorical variant of the term where appropriate (eg, age in 10-year bins) and a 3-knot non-linear restricted cubic spline were compared using analysis of variance testing, with a significance threshold of 5%.

#### Sensitivity analysis

Several additional sensitivity analyses were carried out based on propensity score matching, multiple imputation, and casewise deletion of missing cases. The methods and results are described in the online supplemental material.

## Results

### Clinical characteristics

**Figure 1:**
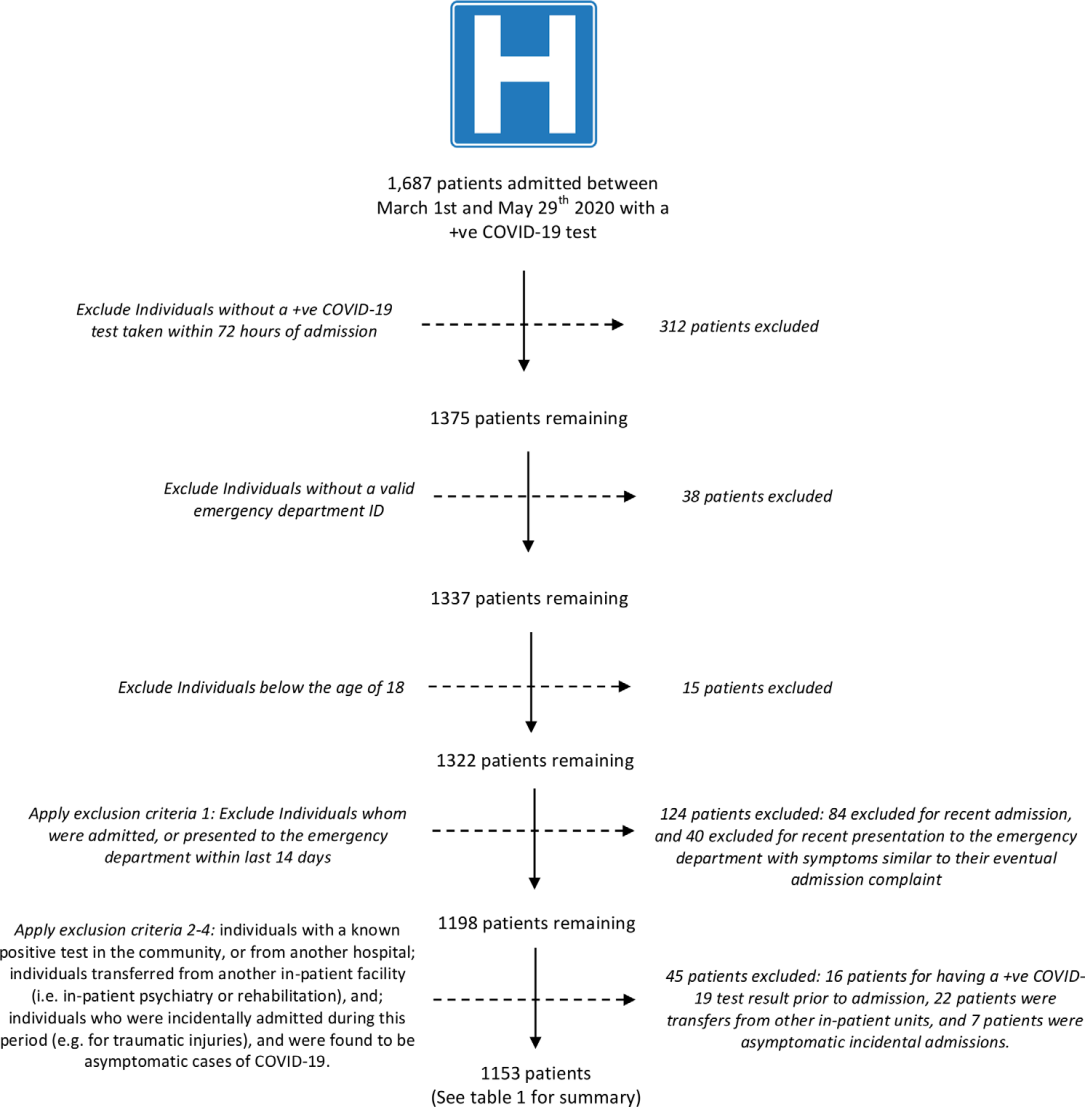
Flow chart of cohort accrual. The flow chart shows the application of the inclusion and exclusion criteria applied to all admissions, resulting in the eventual 1153 patients included in the analysis.

A total of 1153 patients were included in this retrospective observational cohort. Figure 1 summarises the impact of the inclusion and exclusion criteria on the eventual sample size. The baseline characteristics stratified by presence of diarrhoea and exposure to antibiotics prior to admission can be found in table 1. In total, 97 422 patient-days were observed between the start of the observation period and the last date of follow-up. During this period, there were 362 deaths (31.4% mortality), 191 critical care admissions (16.6%), and 155 patients who received mechanical ventilation (13.4%). This equates to a mortality rate of 3.72 per 1000 patient-days. Notably, as 11.9% of the deaths occurred following discharge or transfer, an assumption of survival following discharge as is done in other studies would have resulted in a reported in-hospital mortality rate of 3.32 per 1000 patient-days.

**Table 1:** Recorded characteristics by presence of preadmission diarrhoea

	No diarrhoea (n=932)		Diarrhoea (n=221)	
	No antibiotic exposure prior to admission (n=743)	Antibiotic exposure prior to admission (n=189)	No antibiotic exposure prior to admission (n=190)	Antibiotic exposure prior to admission (n=31)
Age group				
18–24	8 (1.1%)	1 (0.5%)	0 (0.0%)	0 (0.0%)
25–34	15 (2.0%)	2 (1.1%)	5 (3.6%)	1 (3.2%)
35–44	33 (4.4%)	12 (6.3%)	18 (9.5%)	1 (3.2%)
45–54	101 (13.6%)	17 (9.0%)	35 (18.4%)	5 (16.1%)
55–64	141 (19.0%)	32 (16.9%0	44 (23.2%)	4 (12.9%)
65–74	128 (17.2%)	35 (18.5%)	35 (18.4%)	11 (35.5%)
75–84	168 (22.6%)	44 (23.3%)	31 (16.3%)	5 (16.1%)
85–100	149 (20.1%)	46 (24.3%)	22 (11.6%)	4 (12.9%)
Gender				
Female	284 (38.2%)	89 (47.1%)	81 (42.6%)	18 (58.1%)
Male	459 (61.8%)	100 (52.9%)	109 (57.4%)	13 (41.9%)
Ethnicity				
White	329 (44.3%)	77 (40.7%)	92 (48.4%)	20 (64.5%)
Black	233 (31.4%)	58 (30.7%)	50 (26.3%)	5 (16.1%)
Asian	30 (4.0%)	11 (5.8%)	7 (3.7%)	2 (6.5%)
Mixed	10 (1.3%)	3 (1.6%)	2 (1.1%)	0 (0.0%)
Other	47 (6.3%)	12 (6.3%)	11 (5.8%)	2 (6.5%)
Missing	94 (12.7%)	28 (14.8%)	28 (14.7%)	2 (6.5%)
Comorbidities				
Hypertension	431 (58.0%)	118 (62.4%)	101 (53.2%)	17 (54.8%)
Ischaemic heart disease	68 (9.2%)	19 (10.1%)	16 (8.4%)	4 (12.9%)
Congestive heart failure	104 (14.0%)	28 (14.8%)	20 (10.5%)	6 (19.4%)
Cerebrovascular disease	124 (16.7%)	36 (19.0%)	24 (12.6%)	5 (16.1%)
Dementia	130 (17.5%)	48 (25.4%)	17 (8.9%)	2 (6.5%)
Chronic pulmonary disease	197 (26.5%)	77 (40.7%)	44 (23.2%)	11 (35.5%)
Diabetes	257 (34.6%)	57 (30.2%)	63 (33.2%)	9 (29.0%)
Chronic renal disease	135 (18.2%)	41 (21.7%)	35 (18.4%)	6 (19.4%)
Previous or active malignancy	105 (14.1%)	27 (14.3%)	19 (10.0%)	5 (16.1%)
Moderate-severe chronic liver disease	7 (0.9%)	3 (1.6%)	2 (1.1%)	4 (12.9%)
Body mass index (BMI)				
Normal weight (18.5–24.9)	161 (21.7%)	40 (21.2%)	23 (12.1%)	8 (25.8%)
Underweight (<18.5)	26 (3.5%)	11 (5.8%)	6 (3.2%)	0 (0.0%)
Overweight (25.0–29.9)	128 (17.2%)	33 (17.5%)	34 (17.9%)	9 (29.0%)
Medically obese class 1 (30.0–34.9)	82 (11.0%)	17 (9.0%)	31 (16.3%)	5 (16.1%)
Medically obese class 2 (35.0–39.9)	41 (5.5%)	8 (4.2%)	12 (6.3%)	1 (3.2%)
Medically obese class 3 (>39.9)	36 (4.8%)	7 (3.7%)	8 (4.2%)	1 (3.2%)
Missing	269 (36.2%)	73 (38.6%)	76 (40.0%)	7 (22.6%)
Severity markers, median (IQR)*				
Initial NEWS 2	3.0 (2.0–5.0)	3.0 (2.0–6.0)	3.0 (2.0–5.0)	3.0 (1.0–5.0)
Lymphocyte count	0.99 (0.70–1.35)	0.96 (0.68–1.34)	1.0 (0.7–1.3)	1.0 (0.6–1.5)
Neutrophil count	5.6 (3.9–7.9)	6.1 (4.1–8.4)	5.2 (3.9–7.3)	5.0 (4.2–8.0)
Neutrophil:lymphocyte ratio	5.4 (3.4–9.1)	5.9 (3.9–9.5)	5.2 (3.4–7.9)	6.2 (3.7–9.6)
Platelets	213 (163–266)	211 (165–260)	208 (165–263)	231 (155–308)
Creatinine	93 (72–134)	95 (72–139)	90 (47–151)	94 (69–149)
Urea	7.1 (4.7–12.0)	8.2 (5.0–14.4)	6.5 (4.4–10.1)	7.8 (5.5–13.0)
CRP	84 (37–154)	102 (48–169)	90 (47–151)	86 (45–123)
Index of Multiple Deprivation Score, median (IQR)	23.6 (12.2–32.2)	20.5 (11.5–31.4)	22.4 (14.4–31.3)	20.0 (11.3–30.8)
Outcomes				
Critical care admission	123 (16.6%)	35 (18.5%)	29 (15.3%)	4 (12.9%)
Mechanical ventilation	99 (13.3%)	32 (16.9%)	22 (11.6%)	2 (6.5%)
Mortality	231 (31.1%)	78 (41.3%)	46 (24.7%)	7 (22.6%)

*Data missing for 18 patients in total: initial NEWS 2 was missing for n=1; lymphocyte result missing for n=3; neutrophil count missing for n=3; neutrophil-lymphocyte ratio missing for n=3; platelets missing for n=2; creatinine missing for n=1; urea missing for n=8; CRP missing for n=5. Index of Multiple Deprivation missing for n=1. Data are counts and proportions, unless otherwise stated.

CRP, C-reactive protein; NEWS, National Early Warning Score.

### Diarrhoea is not an independent prognostic factor for mortality in COVID-19

A total of 221 patients (19.2%) reported diarrhoea as a symptom prior to admission and confirmation of their COVID-19 status. Patients reporting diarrhoea, compared with those without, were younger (mean age 64.2±15.8 vs 69±16.7 years, p<0.001); less likely to have dementia (unadjusted OR 0.40, 95% CI 0.24 to 0.66); and less likely to have been exposed to antibiotics prior to admission (unadjusted OR 0.64, 95% CI 0.42 to 0.97). Unadjusted outcome risks across the two groups indicated that the presence of diarrhoea was associated with a lower all-cause mortality rate (OR 0.65, 95% CI 0.47 to 0.91). However, diarrhoea was not associated with a significant difference in critical care admission (unadjusted OR 1.03, 95% CI 0.70 to 1.52), or mechanical ventilation (unadjusted OR 0.75, 95% CI 0.47 to 1.18). The summary characteristics and between-group comparisons (diarrhoea vs no diarrhoea) are summarised in online supplemental sTable 1.

Plotting the Kaplan-Meier survival functions illustrates that reporting diarrhoea on index presentation is associated with reduced mortality risk (online supplemental sFigure 1, log-rank test p<0.001). Similar results were seen prior to sequential adjustment of the CPH models (table 2; HR 0.70, 95% CI 0.53 to 0.94, p=0.018). However, following sequential adjustment the point estimates were substantially attenuated, and the overall result was highly non-significant. Results were also non-significant both with adjustment for ethnicity and BMI (HR for diarrhoea: 0.96, 95% CI 0.70 to 1.32, p=0.813), and without adjustment (HR for diarrhoea: 0.95, 95% CI 0.70 to 1.29, p=0.761). A summary of the sequential adjustments and their corresponding results can be found in table 2.

**Table 2:** Sequentially adjusted HRs for diarrhoea, antibiotic use prior to admission, and the diarrhoea:antibiotic interaction term with respect to the primary outcome of all-cause mortality for people admitted with COVID-19

Adjustment	Diarrhoea	Antibiotic use prior to admission	Diarrhoea:antibiotic interaction terms	
			No diarrhoea:antibiotic*	Diarrhoea:antibiotic*
Unadjusted	0.70 (0.53–0.94), p=0.018	1.42 (1.11–1.81), p=0.005	1.47 (1.14–1.90), p=0.003	0.90 (0.41–2.00), p=0.804
Age	0.88 (0.66–1.18), p=0.397	1.28 (1.00–1.63), p=0.049	1.35 (1.05–1.75), p=0.021	0.78 (0.35–1.73), p=0.542
Age and gender	0.92 (0.69–1.23), p=0.564	1.33 (1.04–1.69), p=0.023	1.41 (1.09–1.82), p=0.010)	0.93 (0.37–1.83), p=0.635
Age, gender, and Index of Multiple Deprivation	0.92 (0.68–1.23), p=0.558	1.34 (1.05–1.71), p=0.020	1.41 (1.09–1.83), p=0.009	0.83 (0.38–1.85), p=0.655
Age, gender, Index of Multiple Deprivation and comorbidities†	0.96 (0.71–1.23), p=0.773	1.33 (1.03–1.70), p=0.026	1.40 (1.08–1.82), p=0.012	0.84 (0.37–1.87), p=0.665
Age, gender, Index of Multiple Deprivation, comorbidities† and severity markers‡	0.95 (0.70–1.29), p=0.761	1.24 (0.96–1.60), p=0.098	1.30 (1.00–1.70), p=0.050	0.79 (0.34–1.80), p=0.571
Age, gender, Index of Multiple Deprivation, comorbidities†, severity markers‡ and ethnicity	0.96 (0.70–1.30), p=0.772	1.22 (0.94–1.57), p=0.135	1.37 (0.97–1.66), p=0.080	0.81 (0.36–1.87), p=0.636
Age, gender, Index of Multiple Deprivation, comorbidities†, severity markers‡ and BMI§	0.96 (0.71–1.31), p=0.808	1.18 (0.92–1.53), p=0.195	1.22 (0.94–1.60), p=0.141	0.86 (0.37–2.00), p=0.719
Full covariate set adjustment	0.96 (0.70–1.32), p=0.813	1.17 (0.91–1.51), p=0.231	1.20 (0.92–1.57), p=0.187	0.92 (0.39–2.14), p=0.839

*The base model for the interaction term consisted of both the diarrhoea primary term and the diarrhoea:antibiotic interaction term; the introduction of the interaction term to the primary diarrhoea term improves the Akaike information criterion (AIC) from 4985.42 to 4981.19, but the Bayesian information criterion (BIC) deteriorates from 4988.23 to 4992.87.

†Comorbidities included: hypertension, ischaemic heart disease, congestive heart failure, cerebrovascular disease, dementia, chronic pulmonary disease, diabetes, chronic renal disease, previous or active malignancy, and moderate-severe chronic liver disease.

‡Severity markers included: C-reactive protein (CRP), urea, creatinine, platelet count, neutrophil count, neutrophil:lymphocyte ratio, lymphocyte count, and initial National Early Warning Score 2 (NEWS 2). All of the severity markers and age were modelled using a 3-knot restricted cubic spline. The Index of Multiple Deprivation was modelled as linear feature. BMI was specified using the categories presented in table 1.

BMI, body mass index.

### Community treatment with antibiotics prior to admission was relatively common

Two hundred and twenty patients (19.1%) reported starting a course of antibiotics in the 2 weeks prior to admission, with 259 courses of antibiotics dispensed. The specific antibiotics and the number of prescriptions that they represented are summarised in table 3. There appear to be two major groups of antibiotics that were prescribed: the commonly used ‘lower respiratory tract infection antibiotics’ (amoxicillin, azithromycin, clarithromycin, and doxycycline) and the commonly used ‘urinary tract infection (UTI)’ antibiotics (nitrofurantoin and trimethoprim). Further exploration illustrated that ‘UTI antibiotics’ were more commonly prescribed in older patients (mean age 75.9±15.2 vs 67.9±17.1, p=0.015) and in patients with dementia (unadjusted OR 2.92, 95% CI 1.14 to 7.49).

**Table 3:** Community antibiotic prescriptions for the 211 patients exposed in the 2 weeks prior to admission with COVID-19

	Number of prescriptions dispensed (%)
One antibiotic (n=187)	
Amoxicillin	56 (25.3)
Azithromycin	1 (0.5)
Cephalexin	4 (1.8)
Clarithromycin	15 (6.8)
Co-amoxiclav	27 (12.2)
Doxycycline	24 (10.9)
Erythromycin	1 (0.5)
Flucloxacillin	6 (2.7)
Metronidazole	1 (0.5)
Nitrofurantoin	23 (10.4)
Trimethoprim	4 (1.8)
Unknown	25 (11.3)
Two antibiotics (n=30)	
Amoxicillin+clarithromycin	6 (2.7)
Amoxicillin+co-amoxiclav	3 (1.4)
Amoxicillin+doxycycline	5 (2.3)
Amoxicillin+flucloxacillin	2 (0.9)
Amoxicillin+nitrofurantoin	4 (1.8)
Co-amoxiclav+nitrofurantoin	3 (1.4)
Co-amoxiclav+ciprofloxacin	1 (0.5)
Co-amoxiclav+clarithromycin	1 (0.5)
Co-amoxiclav+doxycycline	1 (0.5)
Co-amoxiclav+erythromycin	1 (0.5)
Co-amoxiclav+flucloxacillin	1 (0.5)
Doxycycline+ciprofloxacin	1 (0.5)
Doxycycline+clarithromycin	1 (0.5)
Three or more antibiotics (n=4)	
Amoxicillin+clarithromycin+metronidazole	1 (0.5)
Amoxicillin+clarithromycin+nitrofurantoin	1 (0.5)
Co-amoxiclav+clarithromycin+doxycycline	1 (0.5)
Co-amoxiclav+ciprofloxacin+nitrofurantoin	1 (0.5)

### Antibiotic use is not an independent prognostic factor for mortality in COVID-19

The summary characteristics and between-group comparisons (antibiotics vs no antibiotics) are summarised in online supplemental sTable 2. Briefly, antibiotic exposure was significantly associated with presence of dementia (n=50 (22.7%) vs n=147 (15.8%), p=0.018), chronic pulmonary disease (n=88 (40.0%) vs n=241 (25.8%), p<0.001), and moderate-severe chronic liver disease (n=7 (3.2%) vs n=9 (1.0%), p=0.027). Unadjusted outcome risks across the two groups suggest that the exposure to antibiotics was associated with a higher mortality rate (unadjusted OR 1.48, 95% CI 1.09 to 2.01), but not critical care admission (unadjusted OR 1.11, 95% CI 0.75 to 1.63), or mechanical ventilation (unadjusted OR 1.23, 95% CI 0.81 to 1.85).

Plotting the Kaplan-Meier survival functions illustrates that recent exposure to antibiotics is associated with increased mortality risk (online supplemental sFigure 1, log-rank test p<0.001). Similar results were seen prior to sequential adjustment of the CPH models (table 2; HR 1.42, 95% CI 1.11 to 1.81, p=0.005). Following sequential adjustment, the point estimate was attenuated, and the overall result became non-significant: both with adjustment for ethnicity and BMI (HR for antibiotic exposure: 1.17 (0.91–1.51), p=0.231), and without adjustment (HR for antibiotic exposure: 1.24 (0.96–1.60), p=0.098). A summary of the sequential adjustments and the corresponding results can be found in table 2, and the full model specification can be found in online supplemental sTable 3.

### The absence of diarrhoea in the presence of antibiotic exposure is independently associated with an increased risk of mortality

**Figure 2:**
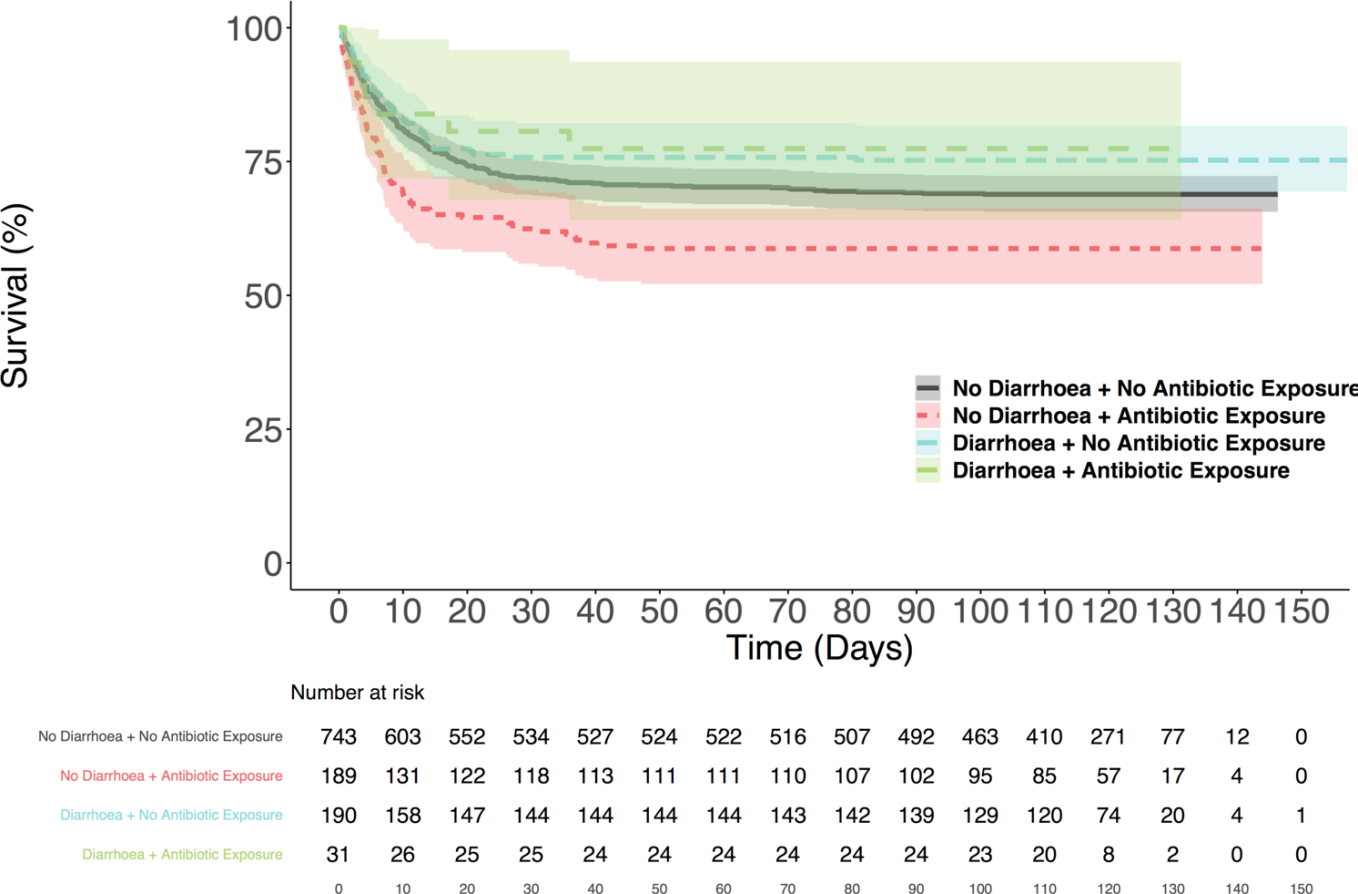
Kaplan-Meier plots for all-cause mortality in 1153 patients admitted with COVID-19, and diarrhoea and exposure to antibiotics prior to admission. The plots show the proportion of patients at risk who were still alive at regular intervals up to 150 days from admission, stratified by the presence of diarrhoea and community exposure to antibiotics. The shaded regions show the 95% CIs.

Exploration of the interaction between the presence of diarrhoea and exposure to antibiotics suggests that there is a significant subgroup effect. Figure 2 illustrates that a subset of patients with antibiotic exposure but no reported diarrhoea appear to have an increased risk of mortality (log-rank test in comparison to all other groups p<0.001); however, none of the other comparisons are statistically significant (p>0.1). Using the interaction term as the primary variable and sequentially adjusting suggests that the aforementioned subgroup effect may not just reflect confounding (adjusted HR from model with all predefined covariate adjustments: 1.30, 95% CI 1.00 to 1.70, p=0.050). However, adjusting for ethnicity and BMI in this setting appears to have a substantial effect on the significance of the result (HR 1.20, 95% CI 0.92 to 1.57, p=0.187). A summary of the sequential adjustments and the results at each stage can be found in table 2, and the fully adjusted models can be found in online supplemental sTable 3.

## Discussion

This analysis of 1153 patients admitted with COVID-19 demonstrates that neither having experienced diarrhoea nor the use of antibiotics in the community (prior to admission) is independently associated with an increased risk of mortality. However, the absence of diarrhoea in patients exposed to antibiotics appears to be associated with a 30% increased risk of mortality, independent of age, gender, IMD, recorded comorbidities, and both biochemical and haematological markers of severity on admission.

### Results in context of the literature

This study confirms previous reports of significant use of antibiotics prior to admission.^21^ In this sample, exposure to community-based antibiotic therapy was associated with being older, identifying as female, and having a diagnosis of dementia, chronic pulmonary disease, or moderate-severe chronic liver disease. However, it is important that these results are interpreted in the appropriate context, as they present a limited perspective on the issue by only including those subsequently admitted to hospital; national testing policy over the course of the first wave of the pandemic meant that individual COVID-19 status for those not admitted was not routinely determined.^22^ This sampling strategy likely explains specific counterintuitive observations,^23^ such as antibiotics being associated with a lower prevalence of diarrhoea. It is more than likely that the overall use of antibiotics was in fact much lower, as a proportion of the total number of cases, and this reflects an important issue for future registry-based research once the linkages between the relevant data sets have been established. An important note for future research is that although not all of the antibiotics noted in our study are those that would be prescribed in presumed respiratory tract infections, the preference for UTI-specific antibiotics in the older patients and those with dementia could reflect the lack of diagnostic clarity often seen in these groups,^24^ and thus it would be important to capture this uncertainty as some of these patients were presumably initially misdiagnosed, or the respiratory coinfection missed.

In light of the substantial community antibiotics therapy observed in this sample, one explanation for aforementioned significant association with mortality risk could be a causal relationship mediated by the gut-lung axis.^25 26^ For example, intestinal involvement in COVID-19 has been shown to downregulate key immune regulators associated with worse outcomes.^27–29^ Moreover, there are microbiome compositions that are more resilient to the effects of antibiotics (in inducing diarrhoea),^30 31^ and are associated with greater host immune system dysfunction via the gut-lung axis.^32 33^ As such, antibiotic-mediated disruption of the gut microbiome and selection for more fastidious organisms could explain the increased risk of mortality observed in this study. However, it is also possible that this association reflects residual confounding, notably the degree of missingness in the ethnicity and BMI variables means that it is impossible to delineate the impact of these features on the exposures of interest, despite the fact that both have been reported to be of particular interest in the context of COVID-19-associated mortality.^34^ Other systematic biases which might explain these results include the potential for the observed exposure (ie, community antibiotics prescription) to be serving as a proxy for some subset of more severe disease not captured by our adjustments. Furthermore, interpretation of these results is complicated by an extensive literature showing improved outcomes with early antibiotic therapy^35^; however, a plausible explanation for the counterintuitive result is that antibiotics were being overprescribed in the community for patients with primarily viral pneumonias (and did not actually have the secondary bacterial pneumonia that the primary care physician was prescribing the antibiotics for). In essence, further research on the effects of antibiotic-mediated changes in gut microbiota on viral respiratory disease is clearly necessary to delineate whether this is likely to be a genuine causal effect. The implications of these investigations will hopefully inform best practice antimicrobial stewardship in the community as they pertain to community-based COVID-19 management.

### Strengths and limitations

The core strength of our study is the use of a robust statistical methodology, employing both sequential adjustment for a range of confounders in combination with an appropriately sized sample, and multiple sensitivity analyses to evaluate the potential influence of missing data; which in combination all lends credibility to our mortality estimates. Moreover, we address a significant limitation of many previous contributions by introducing outcome information that captures postdischarge events, and therefore simplifies the modelling framework as it no longer relies on competing risk assumptions (note: 11.9% of patients who would have been censored under a discharge/transfer as a competing risk approach were actually deceased prior to the end of the follow-up period).

However, given the retrospective observational nature of the study, we were unable to systematically determine whether the diarrhoea that patients reported preceded the antibiotic prescription they received in the community, and as such we were not able to stratify the patients based on the temporal relationship between their symptoms and the potential precipitant (ie, COVID-19 or antibiotic exposure). Furthermore, we were unable to completely eliminate potential bias from other medications such as antimalarials or antiviral drugs which may have been given in the community or within the first 24 hours of admission. These agents were being extensively used in clinical trials at the time, though primarily in hospitalised patients with COVID-19 who were enrolled later in their admission. We have excluded repeat COVID-19 admissions for this reason.

Other limitations include the lack of formal causal inference methodology giving rise to ‘over-adjusted’ results. Moreover, our definition of diarrhoea was left explicitly broad so as not to exclude individuals whose transient experience of this symptom ended prior to admission. As such, it is possible that we captured patients whose experiences do not necessarily fit with well-accepted clinical definitions (such as a combination of Bristol stool scale type 6 or 7 faecal matter and increased stool frequency). This reliance on clinical diagnosis of diarrhoea may also explain some of the associations observed in this study. For example, the lack of diarrhoea reported in patients with dementia may have been due to trouble communicating or fully recalling their symptoms, which would be expected of their underlying pathology. Furthermore, despite having captured a substantial number of postdischarge events, we were not able to cross-reference with the national registry (held by the Office for National Statistics), and as such it is unclear whether our outcome data are truly reflective of all out-of-hospital mortalities.

## Conclusion

This study shows community antibiotic use in patients with COVID-19, prior to hospitalisation, is relatively common, and that neither diarrhoea nor exposure to antibiotics is independently associated with all-cause mortality in COVID-19, after adjusting for confounding factors. However, absence of diarrhoea in antibiotic-exposed patients is associated with increased risk of mortality, although it is unclear whether this represents a causal physiological relationship or residual confounding, therefore meriting further investigation.

## Data Availability

Data are available upon reasonable request. Data cannot be shared publicly due to restrictions associated with access granted to routinely collected information in the absence of informed consent. Requests for access to the data extract used for this study should be directed to kch-tr.cogstackrequests@nhs.net.
