# Supplementary material for: Diarrhoea and preadmission antibiotic exposure in COVID-19: a retrospective cohort study of 1153 hospitalised patients
Source: BMJ Open Gastroenterol. 2021 Sep 6;8(1):e000593. doi: 10.1136/bmjgast-2020-000593 (PMC8423520; doi:10.1136/bmjgast-2020-000593)
Supplement: Supplementary data [file bmjgast-2020-000593supp001.pdf]

Supplementary Material for Gastrointestinal Symptoms & Pre-admission Antibiotic Exposure in COVID-19: a retrospective cohort study of 1153 hospitalised patients

Supplementary Methods

Diagnostic Criteria
Diagnosis of COVID-19 was confirmed by reverse transcriptase polymerase chain reaction (PCR) of nasopharyngeal and/or oropharyngeal swabs or some of respiratory system washing/aspirate as it was the only available method during the study period (i.e. antigen tests has not been approved in the UK by this point). Notably, national testing policy has changed over the course of 2020, however, pillar 2 testing (i.e. community testing) did not begin until 15th April 2020 (and only for healthcare professionals or extremely vulnerable populations such as nursing home residents with dementia). It wasn't until 18th may (10 days prior to the end of the study recruitment period)[1], that testing to opened to all symptomatic individuals. As such, given the dynamic nature of the testing rules, we artificially enforced

Patients with diarrhoea did not routinely had their stool send for culture and microscopy. In the cases where a sample was sent in the accident and emergency department (proportion unquantified), there were 0 cases of infectious diarrhoea or C Difficile identified. One patient tested positive for C. Difficile during the admission, but was negative on admission and thus has been retained in the sample. Local protocols do not include sending stool for faecal calprotectin on presentation with diarrhoea, as such this was not included in the data extraction protocol.

Recruitment

Patients at the recruiting centres whom were included in this study, were also part of the RECOVERY trial, and therefore may have been exposed to specific experimental treatments that altered their clinical course, including Hydroxychloroquine, Dexamethasone, Convalescent Plasma, etc.

Data Extraction from the Electronic Health Records

All of the co-morbidity, biomarker, and demographic data were extracted by the hospital business intelligence unit, based on pre-existing relational databases which aggregate this information following expert-coding for the purposes of submitting remuneration claims against established tariffs. For all included individuals the data was verified, and corrected where necessary, by manual review carried out by 2 clinicians in sequence. Data were collated and checked in a Microsoft excel database. During manual checking of the data, three additional fields were extracted: the initial NEWS2 result on presentation to the emergency department; whether the patient had experienced diarrhoea as part of their illness (documented in the emergency department review, admission clerking, or any entry from the first 24 hours of the admission, and reflecting either a patient-reported phenomenon, or objective finding of Bristol type 6 or 7 stool recorded by a healthcare professional in the Allscripts Bristol stool scale-based chart); exposure to antibiotics in the 14 days preceding admission (either reported by the patient, or identified by a prescription dispensed by their general practitioner which was logged on the local care record which links primary and secondary healthcare service providers in the region, or identified by the admitting pharmacists, who review all admitted patients). Prior to processing, all data was de-identified in line with the UK information commissioner's office (ICO) guidance, and handled in accordance with stipulations prescribed by the King's Electronic Records Research Interface (KERRI) whom provided ethical and data governance-specific oversight to the project.

Censoring at 30 Days Follow-Up

The primary analysis was replicated in full with the follow-up time censored at 30 days to identify whether there was any evidence that the extensive follow-up obscured/biased the effects reported on in the main text.

Sensitivity analysis

Three sensitivity analyses were carried out. The first was based on propensity score match analysis to balance co-variates across those with, and without, diarrhoea. The full set of recorded features, excluding gastrointestinal symptoms, were considered potential confounders and used for matching patients. Missing data was explicitly coded as 'missing' to allow for explicit matching of missingness as well. Matching was carried out based on the logit of the propensity score, a ratio of 3 controls to 1 exposed patient, using a caliper size of 0.05, and without replacement. The next two sensitivity analyses were based on replication in full of the primary analysis using complete datasets (not including the missingness in the ethnicity or BMI fields), created through case-wise

[1] https://www.health.org.uk/publications/long-reads/nhs-test-and-trace-the-journey-so-far

deletion, or multiple imputation. In the latter case, the chained equations approach was utilized, with 5 imputations carried out, based on an assumption of missingness at random.

Methods Not Applied

As described in the main text, Table 2 appears to confirm a significant interaction between diarrhoea and antibiotic use. This leads to several natural questions, which we a priori (having seen the sample sizes) decided not to explore due to sample size constraints. To be clear, the authors are completely blind to the results of any formal analysis of the subsequent questions. The first is whether antibiotic exposure was an explanatory factor in the diarrhoea (e.g. using a logistic regression to allow for adjustments), which would have been an exploration with only 31 events. Moreover, given our eventual results are more pertinent to those who didn't develop diarrhoea, we hope that readers agree it is reasonable not present these (likely mis-fitted) results. Moreover, it is often seen as convention to re-apply models to the stratified datasets when an significant interaction is identified, in an attempt to further unravel the association. Again, the sample sizes are not sufficiently large to suggest that any subsequent results are likely to be robust, and thus we have err-ed on the side of caution by not undertaking them!

Supplementary Results

Community treatment with antibiotics prior to admission was relatively common

The antibiotics in the 31 cases where the individual reported both exposure and diarrhoea on presentation included: Amoxicillin (n = 12), Doxycycline (n = 5), Flucloxacillin (n = 2), Nitrofurantoin (n = 2), Clarithromycin (n = 1), Co-amoxiclav (n =1), Unknown (n = 1), Combinations: Amoxicillin + 1 other (n = 4), Co-amoxiclav + 1 other (n = 2), and Clarithromycin + Doxycyline (n =1).

Sensitivity analyses were consistent with the primary analysis

Propensity score matching (to balance covariates between the diarrhoea and no diarrhoea groups) produced a sample of 791 individuals, with 215 having reported diarrhoea, and 193 mortality events. Replication of the multivariable analysis for both the diarrhoea and diarrhoea:antibiotic interaction term provided similar results to the above: HR for interaction term from adjustment for all covariates other than ethnicity and BMI = 1.58 (95% CI: 1.03 – 2.41; p = 0.035), and HR for diarrhoea term from adjustment for all covariates other than ethnicity and BMI = 1.03 (95% CI: 0.737 – 1.44; p = 0.865). Multiple imputation and case-wise deletion to produce complete datasets resulted in almost identical estimates to the primary analysis as only 17 individuals had missing fields; these results have not been reported in full for the purposes of brevity.

Censoring at 30 Days Follow-Up

As demonstrated by sTable 4 and sFigure 2, the analysis with follow-up censored at 30 days is consistent with the primary analysis, i.e. the interaction between antibiotic exposure and the absence of diarrhoea appears to be associated with an increased risk of mortality – note that the confidence interval for the hazard ratio following the pre-specified adjustments includes 1.00, and thus the p-value falls just above the significance threshold of 5%, however the primary result was borderline as well. Our preferred interpretation is that this demonstrates consistency of a potential effect that requires further investigation.

Table 1: Recorded characteristics of study sample, stratified by presence of diarrhoea as part of complaint

	No Diarrhoea (n = 932)	*Diarrhoea (n = 221)*	*Significance (p-value for 'No Diarrhoea' vs. 'Diarrhoea' Cohort)*
**Age Group**			
18-24	9 (1.0%)	0 (0.0%)	
25-34	17 (1.8%)	6 (2.7%)	
35-44	45 (4.8%)	19 (8.6%)	
45-54	118 (12.7%)	40 (18.1%)	< 0.001
55-64	173 (18.6%)	48 (21.7%)	
65-74	163 (17.5%)	46 (20.8%)	
75-84	212 (22.7%)	36 (16.3%)	
85-100	195 (20.9%)	26 (11.8%)	
**Gender**			
Female			0.222
Male	559 (60.0%)	122 (55.2%)	
**Ethnicity**			
White	406 (43.6%)	112 (50.7%)	
Black	291 (31.2%)	55 (24.9%)	
Asian	41 (4.4%)	9 (4.1%)	0.432
Mixed	13 (1.4%)	2 (0.9%)	
Other	59 (6.3%)	13 (5.9%)	
Missing	122 (13.1%)	30 (13.6%)	
**Co-morbidities**			
Hypertension	549 (58.9%)	118 (53.4%)	0.157
Previous Myocardial Infarction	87 (9.3%)	20 (9.0%)	0.998
Congestive Heart Failure	132 (14.2%)	26 (11.8%)	0.410
Cerebrovascular disease	160 (17.2%)	29 (13.1%)	0.174
Dementia	178 (19.1%)	19 (8.6%)	<0.001
Chronic Pulmonary Disease	274 (29.4%)	55 (24.9%)	0.201
Diabetes	314 (33.7%)	72 (32.6%)	0.814
Chronic Renal Disease	176 (18.9%)	41 (18.6%)	0.986
Previous or Active Malignancy	132 (14.2%)	24 (10.9%)	0.237
Moderate-Severe Chronic Liver Disease	10 (1.1%)	6 (2.7%)	0.120
**Body Mass Index (BMI)**			
Normal Weight (18.5 – 24.9)	201 (21.6%)	31 (14.0%)	
Underweight (<18.5)	37 (4.0%)	6 (2.7%)	
Overweight (25.0 -29.9)	161 (17.3%)	43 (19.5%)	
Medically Obese Class 1 (30.0 – 34.9)	99 (10.6%)	36 (16.3%)	0.075
Medically Obese Class 2 (35.0 – 39.9)	49 (5.3%)	13 (5.9%)	
Medically Obese Class 3 (>39.9)	43 (4.6%)	9 (4.1%)	
Missing	342 (36.7%)	83 (37.6%)	
**Severity Markers (Median [IQR])***			
Initial NEWS 2 Score	3.0 [2.0 – 5.0]	3.0 [2.0 – 5.0]	0.292
Lymphocyte Count	0.99 [0.70 – 1.34]	1.0 [0.70 – 1.36]	0.899
Neutrophil Count	5.7 [3.9 – 8.0]	5.2 [3.9 – 7.5]	0.320
Neutrophil:Lymphocyte Ratio	5.5 [3.5 – 9.3]	5.5 [3.4 – 8.0]	0.136
Platelets	212 [164 – 266]	210 [163 – 260]	0.915
Creatinine	94 [72 – 136]	95 [71 – 130]	0.760
Urea	7.2 [4.8 – 12.2]	6.5 [4.5 – 10.3]	0.114
CRP	87 [37 – 158]	88 [47 – 148]	0.747
**Index of Multiple Deprivation Score (Median [IQR])**	23.2 [12.1 – 32.2]	22.3 [13.5 – 31.0]	0.994
**Antibiotic use prior to Admission**	189 (20.3%)	31 (14.0%)	0.042
**Outcomes**			
Critical Care Admission	158 (17.0%)	33 (14.9%)	0.531
Mechanical Ventilation	131 (14.1%)	24 (10.9%)	0.253
Mortality	309 (33.2%)	54 (24.4%)	0.015

*Legend: * Data missing for 18 individuals in total: Initial NEWS 2 score was missing for n =1; Lymphocyte result missing for n=3; Neutrophil count missing for n = 3; Neutrophil-lymphocyte ratio missing for n =3; Platelets missing for n =2; Creatinine missing for n = 1; Urea missing for n = 8; CRP missing for n = 5. Index of Multiple deprivation missing for n = 1. Data are counts and proportions, unless otherwise stated.*

sTable 2: Recorded characteristics of study sample, stratified by antibiotic exposure status

**Age Group**	*No Antibiotics (n =933)*	*Antibiotics (n = 220)*	*Significance (p-value for 'No Antibiotics vs. 'Antibiotics Cohort)*
18-24	8 (0.9%)	1 (0.5%)	
25-34	20 (2.1%)	3 (1.4%)	
35-44	51 (5.5%)	13 (5.9%)	0.346 (however continuous variable is significant with p value of 0.029)
45-54	136 (14.6%)	22 (10.0%)	
55-64	185 (19.8%)	36 (16.4%)	
65-74	163 (17.5%)	46 (20.9%)	
75-84	199 (21.3%)	49 (22.3%)	
85-100	171 (18.3%)	50 (22.7%)	
**Gender**			
Female			0.012
Male	568 (60.9%)	113 (51.4%)	
**Ethnicity**			
White	421 (45.1%)	97 (44.1%)	
Black	283 (30.3%)	63 (28.6%)	
Asian	37 (4.0%)	13 (5.9%)	0.873
Mixed	12 (1.3%)	3 (1.4%)	
Other	58 (6.2%)	14 (6.4%)	
Missing	122 (13.1%)	30 (13.6%)	
**Co-morbidities**			
Hypertension	532 (57.0%)	135 (61.4%)	0.272
Ischemic Heart Disease	84 (9.0%)	23 (10.5%)	0.590
Congestive Heart Failure	124 (13.3%)	34 (15.5%)	0.465
Cerebrovascular disease	148 (15.9%)	41 (18.6%)	0.369
Dementia	147 (15.8%)	50 (22.7%)	0.018
Chronic Pulmonary Disease	241 (25.8%)	88 (40.0%)	<0.001
Diabetes	320 (34.3%)	66 (30.0%)	0.256
Chronic Renal Disease	170 (18.2%)	47 (21.4%)	0.329
Previous or Active Malignancy	124 (13.3%)	32 (14.5%)	0.704
Moderate-Severe Chronic Liver Disease	9 (1.0%)	7 (3.2%)	0.027
**Body Mass Index (BMI)**			
Normal Weight (18.5 – 24.9)	184 (19.7%)	48 (21.8%)	
Underweight (<18.5)	32 (3.4%)	11 (5.0%)	
Overweight (25.0 -29.9)	162 (17.4%)	42 (19.1%)	
Medically Obese Class 1 (30.0 – 34.9)	113 (12.1%)	22 (10.0%)	0.696
Medically Obese Class 2 (35.0 – 39.9)	53 (5.7%)	9 (4.1%)	
Medically Obese Class 3 (>39.9)	42 (4.7%)	8 (3.6%)	
Missing	345 (37.0%)	80 (36.4%)	
**Severity Markers (Median [IQR])***			
Initial NEWS 2 Score	3.0 [2.0 – 5.0]	3.0 [2.0 – 5.0]	0.110
Lymphocyte Count	1.00 [0.70 – 1.34]	0.96 [0.65 – 1.38]	0.702
Neutrophil Count	5.5 [3.9 – 8.7]	5.9 [4.1 – 8.4]	0.052
Neutrophil:Lymphocyte Ratio	5.4 [3.4 – 8.9]	6.0 [3.8 – 9.6]	0.089
Platelets	212 [164 – 265]	212 [163 – 269]	0.731
Creatinine	94 [72 – 132]	94 [71 – 140]	0.785
Urea	7.0 [4.6 – 11.2]	7.9 [5.1 – 14.3]	0.027
CRP	86 [39 – 153]	98 [48 – 169]	0.069
**Index of Multiple Deprivation Score (Median [IQR])**	23.5 [12.3 – 32.2]	20.4 [12.5 – 31.2]	0.099
**Outcomes**			
Critical Care Admission	152 (16.3%)	39 (17.7%)	0.679
Mechanical Ventilation	121 (13.0%)	34 (15.5%)	0.388
Mortality	278 (29.8%)	85 (38.6%)	0.014

*Legend: * Data missing for 18 individuals in total: Initial NEWS 2 score was missing for n =1; Lymphocyte result missing for n=3; Neutrophil count missing for n = 3; Neutrophil-lymphocyte ratio missing for n =3; Platelets missing for n =2; Creatinine missing for n = 1; Urea missing for n = 8; CRP missing for n = 5. Index of Multiple deprivation missing for n = 1. Data are counts and proportions, unless otherwise stated.*

sTable 3: Hazard ratios and 95% CIs for full covariate set in multivariable analysis of all-cause mortality

	Diarrhoea Model	*Antibiotic Model*	*Diarrhoea:Antibiotics Interaction Model*
**Diarrhoea**			
No	Reference (1.00)	NA	Reference (1.00)
Yes	0.96 (0.70 – 1.32)	NA	1.01 (0.72 – 1.42)
**Antibiotics prior to admission**			
No	NA	Reference (1.00)	NA
Yes	NA	1.17 (0.90 – 1.51)	NA
**Diarrhoea:Antibiotics Interaction**			
No Diarrhoea:No Antibiotic	NA	NA	Reference (1.00)
No Diarhoea:Antibiotic	NA	NA	1.20 (0.92 – 1.57)
Diarhoea:Antibiotic	NA	NA	0.92 (0.39 – 2.14)
**Age**			
Age Spline	1.06 (1.03 – 1.09)	1.06 (1.03 – 1.08)	1.06 (1.03 – 1.08)
Age Spline'	0.98 (0.95 – 1.01)	0.98 (0.96 – 1.01)	0.98 (0.96 – 1.01)
**Gender**			
Female	Reference (1.00)	Reference (1.00)	Reference (1.00)
Male	1.26 (0.98 – 1.62)	1.28 (1.00 – 1.65)	1.27 (0.99 – 1.64)
**Ethnicity**			
White	Reference (1.00)	Reference (1.00)	Reference (1.00)
Black	1.04 (0.75 – 1.43)	1.03 (0.75 – 1.41)	1.02 (0.74 – 1.41)
Asian	1.96 (1.19 – 3.21)	1.93 (1.17 – 3.16)	1.91 (1.16 – 3.14)
Mixed	1.65 (0.65 – 4.23)	1.63 (0.64 – 4.17)	1.58 (0.61 – 4.10)
Other	0.85 (0.47 – 1.54)	0.84 (0.47 – 1.53)	0.84 (0.47 – 1.53)
Missing	1.24 (0.85 – 1.82)	1.23 (0.85 – 1.80)	1.22 (0.83 – 1.78)
**Co-morbidities (versus no)**			
Hypertension	1.11 (0.85 – 1.44)	1.10 (0.85 – 1.44)	1.10 (0.85 – 1.44)
Ischemic Heart Disease	0.99 (0.70 – 1.41)	0.99 (0.70 – 1.40)	0.99 (0.70 – 1.40)
Congestive Heart Failure	1.20 (0.89 – 1.60)	1.20 (0.89 – 1.60)	1.20 (0.90 – 1.61)
Cerebrovascular disease	0.94 (0.71 – 1.23)	0.93 (0.71 – 1.23)	0.93 (0.71 – 1.22)
Dementia	1.85 (1.40 – 2.45)	1.86 (1.41 – 2.46)	1.86 (1.41 – 2.46)
Chronic Pulmonary Disease	1.18 (0.91 – 1.51)	1.16 (0.90 – 1.50)	1.15 (0.89 – 1.49)
Diabetes	1.00 (0.77 – 1.30)	1.01 (0.78 – 1.33)	1.02 (0.78 – 1.32)
Chronic Renal Disease	1.38 (1.04 – 1.83)	1.39 (1.05 – 1.84)	1.38 (1.04 – 1.83)
Previous or Active Malignancy	1.62 (1.21 – 2.16)	1.63 (1.22 – 2.17)	1.63 (1.22 – 2.18)
Moderate-Severe Chronic Liver Disease	1.62 (0.62 – 4.22)	1.49 (0.57 – 3.87)	1.61 (0.60 – 4.35)
**Body Mass Index (BMI)**			
Normal Weight (18.5 – 24.9)	Reference (1.00)	Reference (1.00)	Reference (1.00)
Underweight (<18.5)	0.85 (0.47 – 1.37)	0.80 (0.48 – 1.37)	0.80 (0.47 – 1.36)
Overweight (25.0 -29.9)	1.08 (0.74 – 1.56)	1.07 (0.74 – 1.55)	1.07 (0.74 – 1.55)
Medically Obese Class 1 (30.0 – 34.9)	0.65 (0.40 – 1.07)	0.66 (0.40 – 1.08)	0.65 (0.40 – 1.08)
Medically Obese Class 2 (35.0 – 39.9)	0.93 (0.47 – 1.84)	0.94 (0.47 – 1.86)	0.92 (0.46 – 1.84)
Medically Obese Class 3 (>39.9)	0.58 (0.23 – 1.47)	0.59 (0.23 – 1.50)	0.59 (0.23 – 1.49)
Missing	1.96 (1.45 – 2.64)	1.96 (1.45 – 2.63)	1.94 (1.44 – 2.62)
**Severity Markers**			
Initial NEWS 2 Score Spline	1.15 (1.00 – 1.32)	1.14 (1.00 – 1.31)	1.14 (1.00 – 1.31)
Initial NEWS 2 Score Spline'	1.06 (0.93 – 1.21)	1.06 (0.93 – 1.22)	1.06 (0.93 – 1.21)
Lymphocyte Count Spline	0.15 (0.05 – 0.45)	0.16 (0.05 – 0.47)	0.16 (0.05 – 0.47)
Lymphocyte Count Spline'	5.63 (2.08 – 15.3)	5.52 (2.04 – 15.0)	5.55 (2.05 – 15.1)
Neutrophil Count Spline	1.38 (1.17 – 1.62)	1.37 (1.16 – 1.61)	1.37 (1.16 – 1.62)
Neutrophil Count Spline'	0.74 (0.63 – 0.88)	0.75 (0.63 – 0.88)	0.75 (0.63 – 0.88)
Neutrophil:Lymphocyte Ratio Spline	0.79 (0.69 – 0.90)	0.79 (0.69 – 0.91)	0.79 (0.69 – 0.91)
Neutrophil:Lymphocyte Ratio Spline'	1.35 (1.14 – 1.60)	1.35 (1.14 – 1.60)	1.35 (1.14 – 1.60)
Platelets Spline	1.00 (0.99 – 1.00)	1.00 (0.99 – 1.00)	1.00 (0.99 – 1.00)
Platelets Spline'	1.00 (1.00-1.01)	1.00 (1.00 – 1.01)	1.00 (1.00 – 1.01)
Creatinine Spline	1.00 (0.99 – 1.01)	1.00 (0.99 – 1.01)	1.00 (0.99 – 1.01)
Creatinine Spline'	1.00 (0.99 – 1.01)	1.00 (0.99 – 1.01)	1.00 (0.99 – 1.01)
Urea Spline	1.09 (1.00 – 1.19)	1.09 (1.00 – 1.18)	1.09 (1.00 – 1.18)
Urea Spline'	0.88 (0.76 – 1.01)	0.88 (0.77 – 1.01)	0.88 (0.77 – 1.01)
CRP Spline	1.00 (1.00 – 1.01)	1.00 (1.00 – 1.01)	1.00 (1.00 – 1.01)
CRP Spline'	1.00 (0.99 – 1.00)	1.00 (0.99 – 1.00)	1.00 (0.99 – 1.00)
**Index of Multiple Deprivation Score**	1.00 (0.98 – 1.01)	1.00 (0.98 – 1.01)	1.00 (0.98 – 1.01)

sFigure 1: Kaplan-Meier plots for all-cause mortality in individuals admitted with COVID-19 and: Diarrhoea (Top), or; Exposure to Antibiotics Prior To Admission (Bottom)

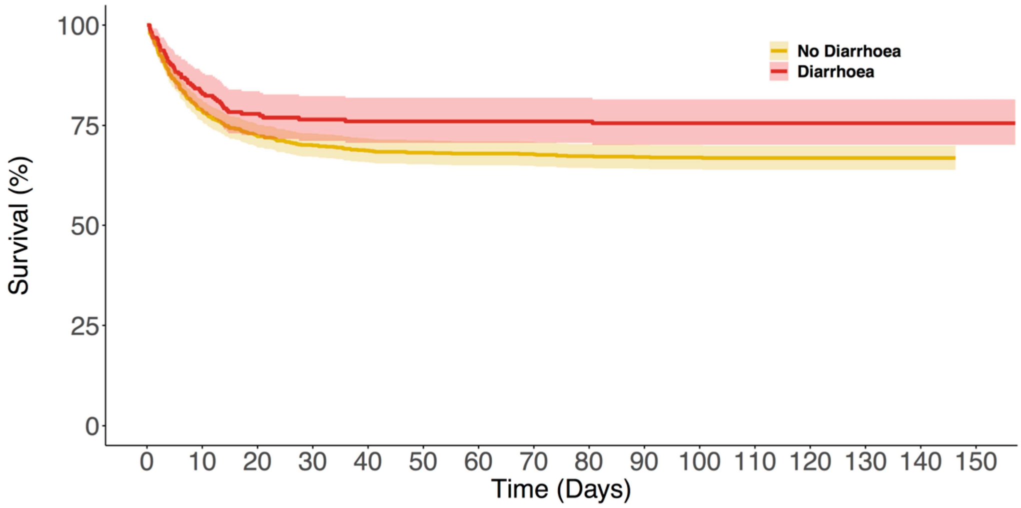

Number at risk

	0	10	20	30	40	50	60	70	80	90	100	110	120	130	140	150
No Diarrhoea	932	734	674	652	640	635	633	626	614	594	558	495	328	94	16	0
Diarrhoea	221	184	172	169	168	168	168	167	166	163	152	140	82	22	4	1

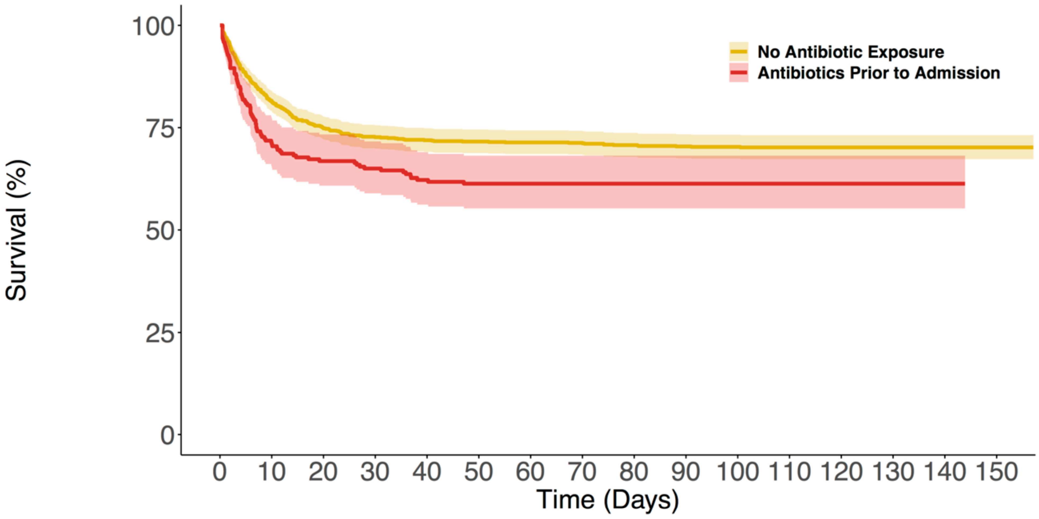

Number at risk

	0	10	20	30	40	50	60	70	80	90	100	110	120	130	140	150
No Antibiotic Exposure	933	761	699	678	671	668	666	659	649	631	592	530	345	97	16	1
Antibiotics Prior to Admission	220	157	147	143	137	135	135	134	131	126	118	105	65	19	4	0

sTable 4: Sequentially adjusted hazard ratios for diarrhoea, antibiotic use prior to admission, and the diarrhoea:antibiotic interaction term with respect to the primary outcome of all-cause mortality for people admitted with COVID-19 (censored at 30 days follow-up).

Adjustment	Diarrhoea	Antibiotic use prior to admission	Diarhoea:Antibiotic Interaction Terms	
			No Diarhoea:Antibiotic*	Diarhoea:Antibiotic*
Unadjusted	0.77 (0.56 – 1.01, p = 0.061	1.39 (1.08 – 1.80), p = 0.011	1.47 (1.12 – 1.93), p = 0.004	0.79 (0.34 – 1.86), p = 0.596
Age	0.95 (0.71 – 1.28), p = 0.750	1.25 (0.97 – 1.61), p = 0.088	1.35 (1.03 – 1.76), p = 0.029	0.68 (0.29 – 1.59), p = 0. 370
Age and gender	0.99 (0.74 – 1.34), p = 0.957	1.30 (1.00 – 1.68), p = 0.046	1.40 (1.07 – 1.83), p = 0.015	0.71 (0.30 – 1.68), p = 0.440
Age, gender, and index of multiple deprivation	0.99 (0.73 – 1.34), p = 0.948	1.31 (1.01 – 1.69), p = 0.041	1.41 (1.07 – 1.85), p = 0.013	0.72 (0.31 – 1.70), p = 0.457
Age, gender, index of multiple deprivation and comorbidities**	1.03 (0.77 – 1.40), p = 0.824	1.03 (1.00 – 1.69), p = 0.048	1.40 (1.06 – 1.84), p = 0.017	0.73 (0.31 – 1.74), p = 0.487
Age, gender, index of multiple deprivation , comorbidities** and severity markers***	1.05 (0.77 – 1.43), p = 0.772	1.20 (0.92 – 1.56), p = 0.183	1.28 (0.97 – 1.70), p = 0.080	0.70 (0.28 – 1.65), p = 0.392
Age, gender, index of multiple deprivation , comorbidities**, severity markers*** and ethnicity	1.04 (0.76 – 1.43), p = 0.811	1.18 (0.90 – 1.55), p = 0.221	1.26 (0.95 – 1.67), p = 0.111	0.71 (0.29 – 1.71), p = 0.441
Age, gender, index of multiple deprivation , comorbidities**, severity markers*** and BMI****	1.08 (0.79 – 1.47), p = 0.651	1.14 (0.87 – 1.49), p = 0.346	1.20 (0.90 – 1.58), p = 0.213	0.75 (0.30 – 1.85), p = 0.530
Full co-variate set adjustment	1.07 (0.77 – 1.47), p = 0.697	1.13 (0.86 – 1.48), p = 0.381	1.17 (0.88 – 1.56), p = 0.266	0.80 (0.32 – 1.98), p = 0.629

*Legend: *The base model for the interaction term consisted of both the diarrhoea primary term, and the diarrhoea:antibiotic interaction term; the introduction of the interaction term to the primary diarrhoea term improves the AIC (Akaike Information Criteria) from 4573.49 to 4569.76, but the BIC (Bayesian Information Criteria) deteriorates from 4577.29 to 4581.18. **Comorbidities included: hypertension, ischemic heart disease, congestive heart failure, cerebrovascular disease, dementia, chronic pulmonary disease, diabetes, chronic renal disease, previous or active malignancy, moderate-severe chronic liver disease. ***Severity markers included: CRP, urea, creatinine, platelet count, neutrophil count, neutrophil:lymphocyte ratio, lymphocyte count, and initial news 2 score. All of the severity markers and age were modelled using a 3 knot restricted cubic spline. The index of multiple deprivation was modelled as linear feature. BMI was specified using the categories presented in table 2.*

sFigure 2: Kaplan-Meier plots for all-cause mortality in 1,153 individuals admitted with COVID-19 and: Diarrhoea and Exposure to Antibiotics Prior to Admission, with follow-up censored at 30 days

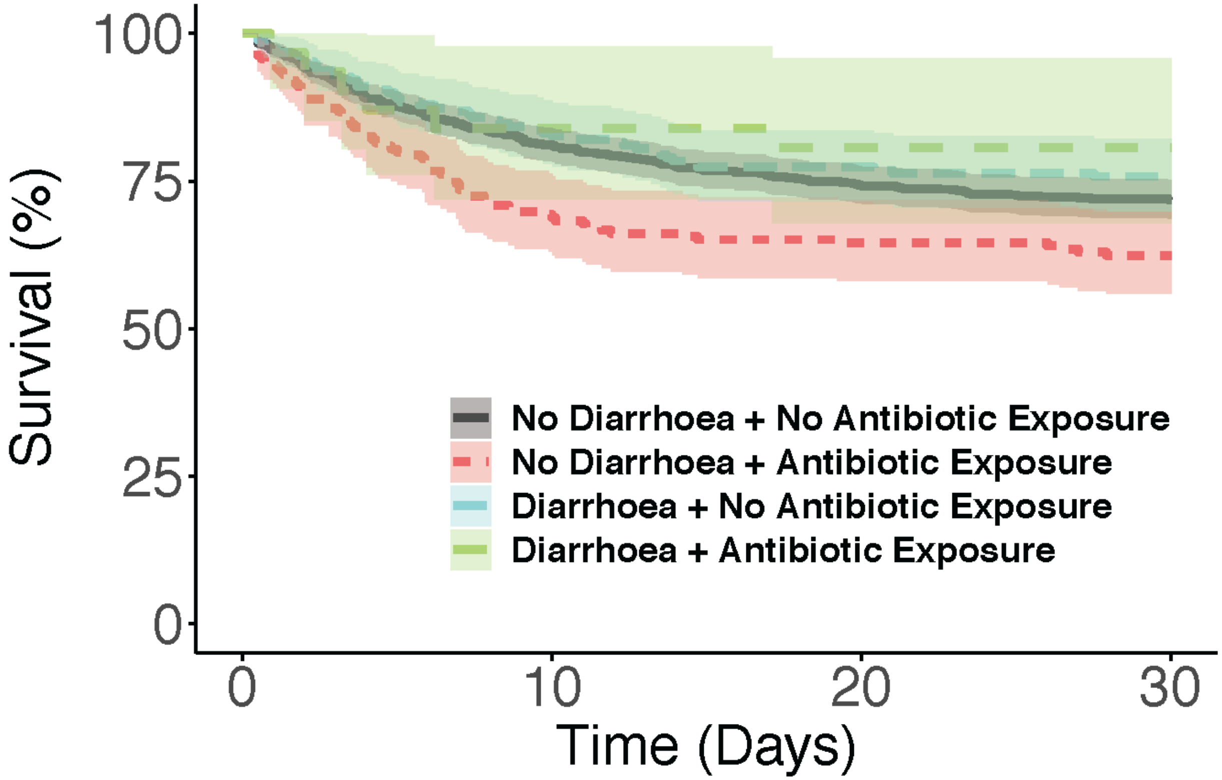

Number at risk

	0	10	20	30
No Diarrhoea + No Antibiotic Exposure	743	603	552	534
No Diarrhoea + Antibiotic Exposure	189	131	122	118
Diarrhoea + No Antibiotic Exposure	190	158	147	144
Diarrhoea + Antibiotic Exposure	31	26	25	25
